# Influence of Dimerization on Aromaticity in Benzene and Heteroaromatic Rings

**DOI:** 10.1021/acs.joc.5c02533

**Published:** 2026-01-13

**Authors:** Jakub Brzeski

**Affiliations:** † Faculty of Chemistry, 49646 University of Gdańsk, Wita Stwosza 63, Gdańsk 80-308, Poland; ‡ QSAR Lab Ltd., Trzy Lipy 3, Gdańsk 80-172, Poland

## Abstract

Noncovalent interactions are known to affect a variety of physicochemical properties. In this study, the effect of their formation between simple aromatic rings such as benzene, pyridine, furan, and pyrrole is analyzed by combining high-level wave functions with energy decomposition analysis and complementary aromaticity metrics. All binding patterns were characterized using the SAPT method, whereas CCSD­(T)-quality wave functions were used for the assessment of most aromaticity indices. Across 13 dimers, dispersion is the most important contributor to the total interaction energy for most motifs. Aromaticity indices based on electron density and geometry change only slightly upon dimerization, indicating that π-delocalization within each monomeric ring is largely preserved. In contrast, NICS(0) values indicate a strong response, especially for π–π bonded systems. These results combine the lack of structural and electronic interference with strong magnetic signatures and provide a reference point for the interpretation of aromaticity descriptors in noncovalent assemblies.

## Graphical abstract

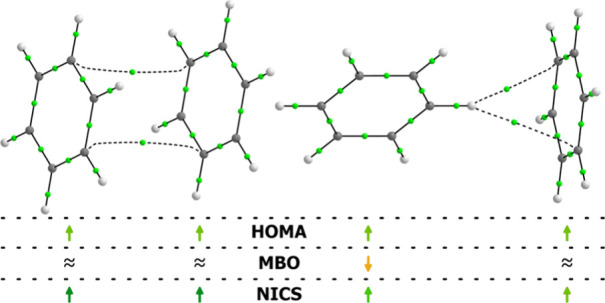

## Introduction

It is widely accepted among scientists that aromaticity is rather ambiguous term.^1^ Since the very beginning of the concept, the level of aromaticity was assessed by means of resonance energy, defined as the difference between the energy of an aromatic compound and that of its olefinic counterpart.^2^ Later on, the aromatic stabilization energy appeared, calculated based on an appropriate homodesmotic reaction.^3^ The said reaction is defined as a reaction in which both sides contain equivalent numbers of carbon atoms in corresponding states of hybridization and the same number of CH bonds.^4^ When it comes to geometrical metrics, Harmonic Oscillator Model of Aromaticity (HOMA) plays a pivotal role.^5^ Built on the differences in bond lengths between a system of interest and suitable fully aromatic molecule, it is a widely used structural indicator of aromaticity and has been used since its initial formulation.^6–12^ The magnetic-based descriptors, such as Nucleus-Independent Chemical Shift (NICS),^13^ are on the other hand based on magnetic shielding.^14–20^ Other important and wide group of aromaticity descriptors is based on electron delocalization. Some of the most important indices within this family are Multicenter bond order (MCBO),^21^ Shannon aromaticity index (SAI),^22^ Para-delocalization index (PDI),^23^ or Aromatic fluctuation index (FLU),^24^ all of which are widely used to assess the aromaticity of a wide range of organic and inorganic systems.^25–35^ Altogether, it is widely accepted that aromaticity is a multidimensional phenomenon.^1,36–38^ Moreover, there is no single metric applicable to a wide range of chemical systems, as the conclusions drawn from various descriptors frequently give conflicting results.^39–43^

Intermolecular interactions are known to affect a plethora of physicochemical properties. Among many others, the representative examples include the following: acidity,^44–46^ boiling points and dielectric constants,^47^ melting and freezing points,^48,49^ viscosity,^50^ surface tension,^51,52^ or solubility.^53,54^ Our recent findings confirm that intermolecular interactions can even affect weights and forms of resonance structures.^55^ The effects of intermolecular interactions on aromaticity remain somewhat underexplored. Nevertheless, several important studies have examined the problem. Namely, Corminboeuf and colleagues have found that the stacking of 4nπ-electron hydrocarbon rings can render otherwise antiaromatic systems through-space aromatic.^56^ Similar conclusions were drawn for cyclobutadiene,^57^ cyclophane,^58^ and porphyrins.^59^ It was found that hydrogen bonding often increases aromaticity for model complexes made of HF as a proton donor and 2-methylene-2*H*-indene derivatives in the paper by Nekoei and Vatanparast.^60^ The study of Krygowski et al. has shown that, for phenol-based H-bonded complexes with various bases, aromaticity decreases as a result of bond elongation.^61^ Shakerzadeh and collaborators have studied the effect of H-bonding on the aromaticity of substituted pentavulfenes using indices like NICS, HOMA, FLU, and SAI. They have found that variations in SAI and HOMA values correlate with the properties of the H-bond. At the same time, the remaining indices were found to lead to inconclusive results.^62^ The effects of cation−π and anion−π interactions have likewise attracted attention. Namely, based on the values of NICS(1) and NICS_ZZ_(1), Rodríguez-Otero et al. have found that complexation of aromatic rings with several ions (Li^+^, Na^+^, K^+^, F^–^, and Cl^–^) leads to only minimal change in aromaticity.^63^

Noncovalent dimerization of aromatic rings is ubiquitous across chemical and biological contexts, shaping assembly and recognition in molecular crystals,^64,65^ proteins,^66–68^ and nucleic acids.^69^ It also underpins asymmetric synthesis,^70^ crystal engineering,^71,72^ and supramolecular recognition.^73^ Although the dimers of popular aromatic rings such as benzene,^74,75^ pyridine,^76,77^ furan,^78^ and pyrrole^79,80^ seem to be well studied from various perspectives, the effect of dimerization on aromaticity remains comparatively underexplored. This work addresses that gap by systematically evaluating changes in aromaticity upon dimerization in these systems.

## Experimental Section

### Theoretical Background

Based on the wave functions constructed from natural orbitals obtained during CCSD­(T) calculations, a set of five aromaticity indices was calculated for both the monomeric and dimeric systems: (i) HOMA, (ii) SAI, (iii) H (total energy density at the ring critical point), (iv) normalized Multicenter Bond Order (nMCBO), and (v) NICS(0) (NICS at the ring center).

Since the establishment of its generalized form in 1993 by Krygowski,^5^ the HOMA index has become the most widely used geometry-based aromaticity index.^81^ The harmonic oscillator model of aromaticity (HOMA) index is an excellent structural indicator of aromaticity.^31–35^ It is calculated as follows:

$$\mathrm{HOMA}=1-\frac{\alpha }{n}\sum (R_{\mathrm{opt}}-R_{ij}{)}^{2}$$

with *n* representing the number of bonds taken into summation, α and *R*_opt_ are normalization and bond constants, respectively, given in the original paper, whereas *R*_*ij*_ is the bond length between atoms *i* and *j* in the structure of interest.^5^ The further the actual bond lengths (*R*_*ij*_) deviate from the ideal (*R*_opt_), the lower the HOMA index values and thus the aromaticity of a given system. The SAI, FLU, MBO, and HOMA calculations were carried out using the Multiwfn package (version 3.8­(dev)).^82^

The SAI is a measure of aromaticity based on Shannon entropy. Its calculation is based on electron densities at bond critical points (BCPs) corresponding to the bonds within the ring of interest. It is calculated as^22^

$$\mathrm{SAI}=\ln(N)-\sum_{i}^{N}(-p_{i}\ln p_{i})$$

where *N* is the number of BCPs in the ring, and *p*_*i*_ represents the normalized electron density at the *i*th BCP.

Total electron energy density (*H*) values corresponding to RCPs were extracted for the assessment of the aromaticity of studied systems according to the procedure proposed by Palusiak and Krygowski.^83^

The MCBO^21^ is a generalization of Mayer bond order to multicenter systems. Its normalized version (nMBO) allows one to compare the aromaticity of rings with different sizes.^84^ Although SAI and nMBO are both based on electron delocalization, they were selected as complementary. SAI quantifies aromaticity based on π-electron delocalization using information theory, and nMCBO measures multicenter bonding. As such, they provide a more complete picture of electronic aromaticity than any of them alone.

NICS­(0) is a widely used computational magnetic descriptor for the assessment of the aromaticity of a given ring. It is defined as the negative of the isotropic magnetic shielding computed at a ghost atom located in the center of a ring. The more negative the NICS(0) values, the stronger the diatropic ring current, which is an indication of higher aromaticity.

## Computational Details

The geometries of four benzene dimers, i.e., T-shaped tilted, T-shaped, parallel-displaced, and sandwich, were taken from the Supporting Information associated with the paper of Hobza and co-workers, where they were optimized at the DFT-D/BLYP/TZVP level parametrized for the benzene dimer to reproduce CCSD­(T)/CBS reference data.^85^ In the present work, these benzene dimer geometries are used without further optimization. For the dimers of heterocyclic rings, as well as for all monomers, the equilibrium geometries were optimized at the MP2 ^86,87^ /aug-cc-pVTZ^88,89^ level. For small neutral complexes, MP2 with a sufficiently large correlation-consistent basis set (e.g., aug-cc-pVTZ) is known to provide both reliable equilibrium geometries and intermolecular distances in good agreement with higher-level methods.^90^ At the same time, the performance of dispersion-corrected DFT highly depends on the specific functional and damping scheme used during calculations.^91,92^ The studied compounds were selected to cover changes in the ring size, heteroatom type, and lone-pair placement. In order to ensure that the stationary points found are minima on the potential energy surface, the optimizations were followed by vibrational analysis. All of the above-mentioned calculations were carried out using the GAUSSIAN16 (Revision C.01) software.^93^ The Cartesian coordinates of the equilibrium structures of the dimers of pyridine, furan, and pyrrole studied in this paper are collected in Table S1. Since the equilibrium structures of the dimers studied here are well-known and extensively discussed elsewhere, they are not treated further in this paper.^94–100^

Previous studies have shown that the potential energy surface of the benzene dimer is exceptionally flat.^101,102^ For that reason, the CCSD­(T)^103,104^ method, together with an augmented correlation-consistent basis set, is necessary for an accurate description of its nuances.^105–107^ Additionally, it was confirmed that the application of the CCSDT­(Q) approach improves the energies of the benzene dimer only by 0.058 kcal/mol.^108^ In an effort to obtain CCSD­(T)-level wave functions, calculations at the CCSD­(T)/aug-cc-pVDZ^88,89^ level were carried out using the Density Fitting approximation for both SCF and CC modules.

The Symmetry-Adapted Perturbation Theory (SAPT)^109^ is a method designed specifically to analyze intermolecular interactions by treating the interaction between two monomers as a perturbation of the isolated systems. It allows one to compute the total interaction energy as well as decompose it into four physically meaningful components, i.e., electrostatics, exchange, induction, and dispersion. In this study, the SAPT calculations with many-body dispersion treatment were conducted at the SAPT2 + 3­(CCD)^110,111^ /aug-cc-pVTZ level together with the assessment of the stabilization energy arising from charge transfer.^112^ SAPT calculations were performed in order to split the total interaction energies into electrostatics, exchange, induction, and dispersion contributions and thus better comprehend the investigated systems. All interaction energy calculations were carried out with the frozen-core approximation. The CCSD­(T) wave function calculations as well as SAPT calculations were carried out using the PSI4 (1.9.1 release) computational package.^113^

The Quantum Theory of Atoms in Molecules (QTAIM)^114^ analysis was carried out to find and characterize the Bond Critical Points (BCPs) corresponding to the intermolecular interactions as well as Ring Critical Points (RCPs) present in the studied dimers. The CCSD­(T)/aug-cc-pVDZ wave function was used as input for all QTAIM calculations. The QTAIM calculations were carried out using the AIMAll software (Version 19.10.12).^115^

The NICS­(0) calculations were carried out using the gauge-including atomic orbitals (GIAO) method,^116^ the Jensen pcSseg-2 basis set, optimized for nuclear magnetic shielding constants,^117^ and the AutoAux auxiliary^118^ basis sets. The computations were conducted at the RI-MP2 level of theory based on relaxed electron densities.^119^ This level of theory has been shown to yield NMR shifts in very good agreement with high-level coupled-cluster reference data.^120,121^ The position of the ghost atom in the calculations was determined as the geometric center of five or six atoms (depending on the system) forming the ring of interest using the formulas $x_{c}=\frac{1}{n}\sum_{i=1}^{n}x_{i},\;y_{c}=\frac{1}{n}\sum_{i=1}^{n}y_{i}$ and $z_{c}=\frac{1}{n}\sum_{i=1}^{n}z_{i}$ . All NICS(0) calculations were carried out with the ORCA (version 6.1) software package.^122^

All CCSD­(T), SAPT, QTAIM, and NICS analyses were performed as single-point calculations based on the MP2/aug-cc-pVTZ (or Hobza DFT-D/BLYP/TZVP for benzene)-optimized geometries.

## Results and Discussion

### Dimer Interaction Energies

The equilibrium structures of all 13 dimers studied here are presented in Figures 1 and 2. They were selected to represent a variety of geometries and noncovalent interaction stabilization mechanisms. Namely, for each system, at least one π–π-stabilized dimer is present. The studied compounds also exhibit diversity when it comes to hydrogen bonding; e.g., the T-shaped tilted isomer of pyrrole is stabilized by an N–H···π interaction, whereas the H-bonded variant of pyridine is held together by the C–H···N interaction.

**Figure 1:**
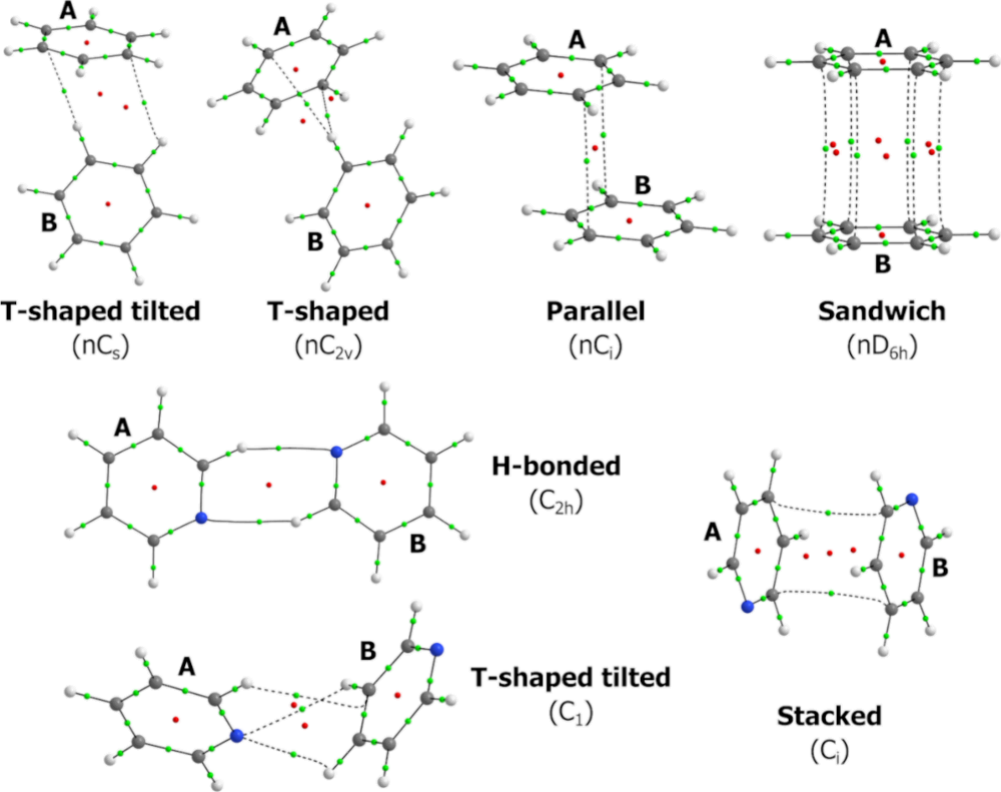
Equilibrium structures of the benzene and pyridine dimers together with the corresponding point groups, bond (green), and ring (red) critical points. The RCP’s corresponding to nonaromatic rings were removed for visibility. The ‘n’ in front of the point groups describing benzene dimers (geometries from paper of Hobza and co-workers^85^) stands for ‘nearly’, as their Cartesian coordinates are very close to the ideal symmetry but were obtained from unconstrained optimizations.

**Figure 2:**
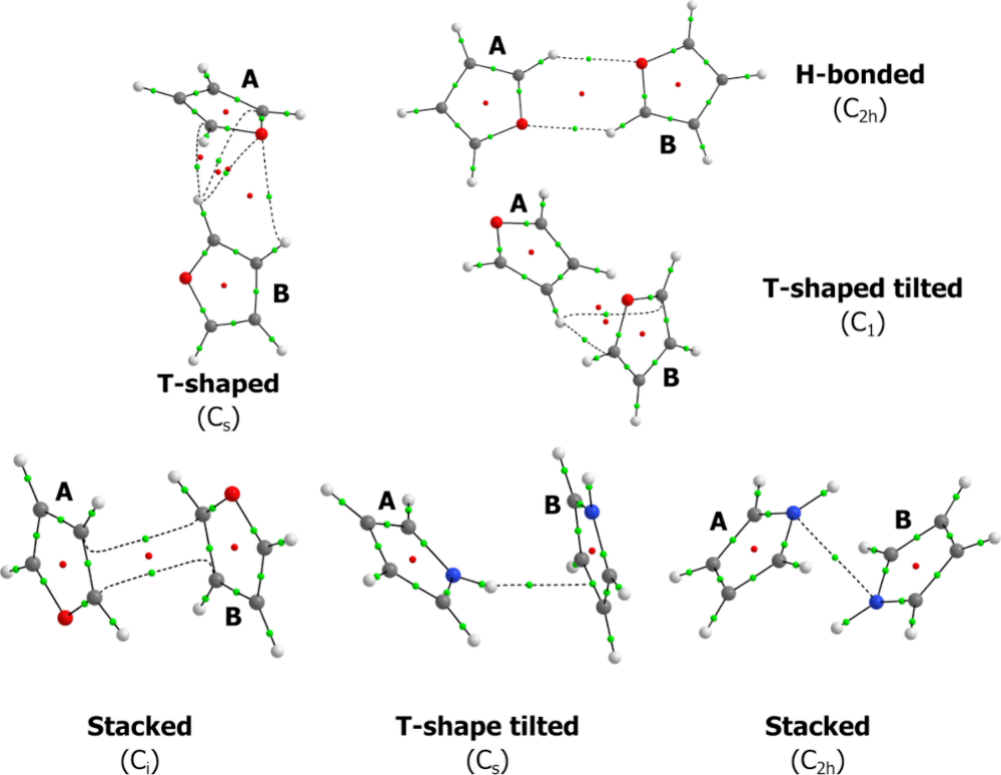
Equilibrium structures of the furan and pyrrole dimers together with the corresponding point groups and bond (green) and ring (red) critical points. The RCP’s corresponding to nonaromatic rings were removed for visibility.

As indicated in Table 1, when it comes to benzene dimers, the SAPT2 + 3­(CCD) interaction energies all fall in the range of −1.5 to −3.0 kcal/mol. The two strongest interactions correspond to the two C–H···π hydrogen-bond stabilized T-shaped conformers with corresponding *E*_int_ calculated to be −2.92 and −2.87 kcal/mol for tilted and regular isomers, respectively. The π–π stacked parallel-displaced conformer was found to be bound by −2.62 kcal/mol, closely matching the stability of the two conformers mentioned above. The *E*_int_ calculated for the sandwich isomer is equal to −1.56 kcal/mol, rendering it the weakest bound out of all benzene conformers studied in this paper. The ordering agrees very well with the findings of Czernek and Brus who calculated the interaction energies of the three most bound isomers at the CCSD­(T)/CBS level to be 2.83, 2.71, and 2.68 kcal/mol for T-shaped tilted, T-shaped, and parallel-displaced conformers, respectively.^75^ Additionally, previous reports have stressed the limited stability of the sandwich isomer.^123^ When it comes to individual contributions, all dimers are dispersion-driven, as even for C–H···π-stabilized systems, the total contribution of dispersion to all attractive interactions is as high as 62–64%. As expected, it is even higher for π–π bonded conformers and via a 77% contribution for the parallel-displaced isomer and rises to 100% for the sandwich conformer. It is worth noting that for the sandwich dimer, the electrostatic and induction contributions are slightly destabilizing, and thus, the geometry is held together only by dispersion. The exchange repulsion was calculated to be 5.67 kcal/mol for the parallel-displaced conformer. This likely arises from significant Pauli repulsion, as the aromatic rings lie relatively close together, closer than they are in, e.g., the sandwich isomer. Both polarization and charge transfer have stabilizing effects in all four isomers. The induction energy in a dimer-centered basis is relatively small, as it does not exceed −0.30 kcal/mol for any of the benzene dimers. In the case of T-shaped systems, most of the induction stabilization comes from polarization, whereas for the π–π systems, charge transfer dominates. This can be elucidated by the fact that stacking allows for better overlap of two π systems and thus coupling between filled π on one ring and virtual π* on the other one. In SAPT terms, larger overlap yields greater off-diagonal Fock matrix elements, which increases the short-range charge transfer.

**Table 1:** SAPT Interaction Energies and Charge-Transfer (CT) Kinetics in Aromatic Dimers^a^

system			SAPT interaction energies					SAPT charge transfer analysis			
aromatic ring	dimer geometry	type of noncovalent interaction	*E*_int_	electrostatics	induction	dispersion	exchange	CT	induction (MB)	induction (DB)	CT contribution to induction (DB)
benzene	T-shaped tilted	C–H···π	–2.92	–1.80 (25.81)	–0.68 (9.72)	–4.50 (64.48)	4.06	–0.09	–0.16	–0.25	36.76
	T-shaped	C–H···π	–2.87	–1.78 (26.33)	–0.77 (11.38)	–4.22 (62.29)	3.90	–0.10	–0.21	–0.30	32.12
	parallel-displaced	π–π	–2.62	–1.36 (16.43)	–0.57 (6.85)	–6.36 (76.73)	5.67	–0.15	–0.06	–0.21	71.66
	sandwich	π–π	–1.56	0.10 (0.00)	0.06 (0.00)	–4.97 (100.00)	3.25	–0.08	–0.03	–0.11	69.23
pyridine	H-bonded	C–H···N	–4.21	–5.62 (48.57)	–1.73 (14.96)	–4.22 (36.47)	7.35	–0.40	–0.88	–1.28	31.47
	T-shaped tilted	C–H···N	–3.52	–4.09 (43.49)	–1.03 (10.93)	–4.29 (45.58)	5.89	–0.19	–0.38	–0.57	33.18
		C–H···π									
	stacked	π–π	–3.30	–5.27 (32.15)	–1.37 (8.36)	–9.75 (59.49)	13.08	–0.41	–0.05	–0.46	89.46
furan	T-shaped	C–H···O	–2.81	–2.84 (33.40)	–0.83 (9.76)	–4.83 (56.85)	5.69	–0.14	–0.24	–0.39	36.70
		C–H···π									
	H-bonded	C–H···O	–2.73	–3.00 (44.69)	–0.74 (10.96)	–2.98 (44.35)	3.98	–0.20	–0.28	–0.49	41.72
	T-shaped tilted	C–H···O	–2.16	–2.49 (30.26)	–0.85 (10.31)	–4.90 (59.43)	6.09	–0.24	–0.09	–0.32	73.38
		C–H···π									
	stacked	π–π	–1.97	–3.99 (32.68)	–1.05 (8.57)	–7.17 (58.74)	10.24	–0.44	0.13	–0.31	141.84
pyrrole	T-shaped tilted	N–H···π	–6.46	–7.86 (43.07)	–3.05 (16.70)	–7.35 (40.24)	11.79	–0.41	–1.51	–1.92	21.49
	stacked	π–π	–6.27	–8.76 (44.15)	–2.34 (11.82)	–8.73 (44.03)	13.56	–0.40	–0.96	–1.36	29.57

a All energies are reported in kcal/mol. For attractive components of the total interaction energy (*E*_int_(SAPT)), the corresponding percentage contributions are given in parentheses. The CT contribution to induction (DB) is reported as a percentage.

In the case of pyridine, each of the three studied conformers is linked via a different mode. Namely, the H-bonded isomer is of C_2h_ symmetry and as such is noncovalently bonded by two equivalent C–H···N contacts. It should be noted that C–H···N dimer stabilization arises from both dipole–dipole and dispersion forces rather than from classical hydrogen bonding. An analogous situation is found for the C–H···O-stabilized dimer of furan, referred to in this paper as H-bonded for short. In Table 1 and both figures, these two dimers are still labeled as H-bonded for brevity and to keep a uniform naming scheme with the remaining dimers. In the T-shaped tilted pyridine dimer, two different hydrogen bonds are present: C–H···π and C–H···N. In the first interaction, the A ring acts as a donor, while the other accepts through its π system. In the second, the A ring contributes a C–H, while the partner ring accepts a C–H through its N atom. As such, this isomer can be thought of as originating from the reorientation of the H-bonded isomer that swaps one C–H···N bond for C–H···π. Altogether, the *E*_int_ calculated for this system is equal to −3.52 kcal/mol. The last of the isomers considered arises from π–π stacking, with nitrogen atoms sitting opposite each other (C_i_) and is bound by 3.30 kcal/mol. For pyridine dimers, the relative energies of the isomers found in this paper are in agreement with earlier reports.^124^ Similarly to what was observed for the benzene dimer, dispersion plays a crucial role in the stabilization of all conformers. Namely, regardless of the binding mode, its contribution to the total attractive interaction is not lower than 36% in any example and reaches as much as 59% for the stacked isomer. The two rings forming the H-bonded isomer interact mainly through electrostatics. Unsurprisingly, the highest exchange repulsion was found for the stacked isomer and amounts to 13.08 kcal/mol. The |CT| is lower than 0.41 kcal/mol in every example. In the case of the H-bonded and T-shaped tilted isomers, charge transfer amounts to 31.37 and 33.18% of induction stabilization. When it comes to the stacked isomer, it is as high as 89.46%.

Out of the four isomers of (C_4_H_4_O)_2_, the three most stable ones are again stabilized by hydrogen bonding, whereas the least stable one arises from π–π stacking. The values collected in Table 1 indicate that the order of stability is as follows: T-shaped ≈ H-bonded > T-shaped tilted > stacked. The corresponding *E*_int_ values span over a range smaller than 1 kcal/mol, i.e., from −2.81 to −1.97 kcal/mol. The T-shaped configuration is stabilized by C–H···O and C–H···π noncovalent interactions, where hydrogen atoms of ring A act as donors in both cases. The H-bonded dimer with C_2h_ symmetry is held together by two equivalent C–H···O interactions, similar to what was observed for (C_5_H_5_N)_2_. In a T-shaped tilted conformer, two BCPs were found (see Figure 2) connecting a single H atom of ring A with ring B both via its O atom and via a C–H···π interaction. The stacked isomer of furan is, analogous to its pyridine counterpart, linked through π–π interactions. The SAPT analysis reveals that dispersion is the dominant factor in the formation of furan dimers in all cases but the H-bonded one, where its contribution (44.35%) is the same as that of the electrostatics (44.69%). Due to the interaction of aromatic rings, the Pauli repulsion has the most destructive effect on the stacked conformer. The highest CT stabilization calculated for any system studied in this paper was obtained for the stacked isomer of furan, with a value of −0.44 kcal/mol. Additionally, in this case, the polarization contribution to the induction is destabilizing (+0.13 kcal/mol). For this reason, the magnitude of CT is larger than that of the final induction, because CT both cancels the repulsive polarization and supplies the net stabilization. For the T-shaped tilted system, CT is the principal contribution to the induction energy, whereas for the remaining two systems, polarization remains the dominant component.

The two pyrrole dimers are the most tightly bonded systems in this study. Namely, the T-shaped tilted N–H···π has an *E*_int_ of −6.46 kcal/mol, whereas the π–π stacked one binds by −6.27 kcal/mol. Electrostatics and Dispersion are again the most important contributor to the overall stability exceeding 40% of total attractive interactions in both examples. Unsurprisingly, due to stacking, the exchange destabilization is larger in the stacked isomer by 1.77 kcal/mol. The CT is almost identical in two cases and amounts to approximately −0.4 kcal/mol stabilization. In both cases, polarization dominates over CT.

### Aromaticity Changes upon Dimerization

Due to the fact that the main aim of this paper is to assess the influence of dimerization on aromaticity, the shift in the aromaticity index within the rings forming the given dimer is presented in the main body of the paper (Table 2). These results are compared with values calculated for isolated monomers, which are collected in Supporting Information (Table S2). In all cases, the change in the value of a given index is defined as Δ*X* = *X*_dimer_ – *X*_monomer_, where the two quantities denote the values of a given index for the ring in the dimer and the isolated monomer, respectively. The nominal values of HOMA, SAI, nMBO, and NICS(0) indices can also be found in Table S2.

**Table 2:** Changes of Aromaticity Indices upon Dimerization^a^

aromatic ring	dimer geometry	ΔHOMA-A	ΔHOMA-B	ΔSAI-A	ΔSAI-B	Δ*H*-A	Δ*H*-B	ΔnMBO-A	ΔnMBO-B	ΔNICS(0)-A	ΔNICS(0)-B
benzene	T-shaped tilted	0.00722	0.00681	7.28 × 10^–8^	2.67 × 10^–7^	–0.00016	0.00006	–0.0070	–0.0222	0.116	–0.410
	T-shaped	0.00700	0.00687	1.08 × 10^–8^	3.75 × 10^–7^	–0.00020	0.00007	–0.0032	–0.0431	–0.013	–0.409
	parallel-displaced	0.00828	0.00829	7.62 × 10^–7^	7.68 × 10^–7^	0.00005	0.00005	–0.0014	–0.0014	–1.137	–1.137
	sandwich	0.00876	0.00876	9.00 × 10^–10^	9.00 × 10^–10^	0.00009	0.00009	0.0239	0.0239	–0.764	–0.764
pyridine	H-bonded	–0.00106	–0.00106	–9.84 × 10^–5^	–9.84 × 10^–5^	–0.00004	–0.00004	0.0241	0.0241	0.232	0.232
	T-shaped tilted	–0.00027	–0.00091	–6.38 × 10^–5^	–1.93 × 10^–5^	–0.00001	–0.00004	0.0411	0.0432	0.088	0.350
	stacked	–0.00056	–0.00056	–1.90 × 10^–5^	–1.90 × 10^–5^	–0.00008	–0.00008	–0.0425	–0.0316	–1.680	–1.680
furan	T-shaped	–0.00061	–0.00361	–9.13 × 10^–6^	–8.05 × 10^–4^	0.00007	0.00007	0.0314	0.0189	–0.397	–0.267
	H-bonded	–0.02952	–0.02952	2.42 × 10^–4^	2.42 × 10^–4^	0.00025	0.00025	–0.0101	–0.0101	0.221	0.221
	T-shaped tilted	–0.00838	0.02225	1.77 × 10^–5^	–1.77 × 10^–4^	–0.00001	–0.00001	0.0619	0.0283	–0.271	–0.686
	stacked	0.01140	0.01140	–8.34 × 10^–5^	–8.34 × 10^–5^	0.00004	0.00004	–0.0120	–0.0120	–1.517	–1.517
pyrrole	T-shaped tilted	0.00971	0.00058	–3.46 × 10^–5^	3.58 × 10^–6^	–0.00012	–0.00009	–0.0244	0.0099	–0.390	–0.737
	stacked	–0.00027	–0.00027	1.85 × 10^–5^	1.85 × 10^–5^	–0.00010	–0.00010	–0.0034	–0.0034	–0.773	–0.773

a Δ*H* values are given in au, ΔNICS(0) values are in ppm, whereas the remaining ones are dimensionless.

When assessed structurally by the corresponding values of ΔHOMA, benzene dimerization seems to lead to a slight increase in aromaticity across all examined systems. The change however is within the numerical noise of both level of theory and geometry convergence thresholds. For the two isomers with asymmetric electron density distribution, i.e., T-shaped tilted and T-shaped, the increase in aromaticity is higher for ring A—acceptor of the hydrogen bond, than it is for ring B—donor of the said bond. Altogether, it can be said that the increase in aromaticity follows the order T-shaped ≈ T-shaped tilted < parallel-displaced < sandwich. The SAI calculated for isolated benzene is equal to 0, which arises from the fact that D_6h_ symmetry and equalized π-bonding make the ring perfectly uniform. This result significantly differs from the 1.7 × 10^–6^ value calculated at the lower level of B3LYP/6-31+G** by Noorizadeh and Shakerzadeh.^22^ Because benzene has SAI = 0, any of its derivatives, whether arising from substitution or, as in this case, from clustering that disrupts its uniform π-electron distribution, will raise the SAI value, indicating lower aromaticity by this index. The lowest value of ΔSAI calculated for any dimer in this paper is equal to 9.00 × 10^–10^ for a sandwich conformer. Values of ΔSAI collected in Table 2 suggest that the aromaticity of (C_6_H_6_)_2_ conforms the same order which was observed for HOMA indices suggesting that π–π stacking increases or in the case of SAI retains aromaticity to a higher degree than hydrogen-bonding. When it comes to Δ*H* values, they seem to indicate the increase in aromaticity of all benzene dimer rings studied except the two accepting the H-bond, i.e., by donating electron density in T-shaped tilted and T-shaped conformers. Slightly positive values of Δ*H* calculated for the remaining rings, including the symmetrical π–π bonded isomers, indicate subtle increase in aromaticity. The ΔnMBO values show results that are similar to what was observed for the SAI values. Namely, the numbers indicate the decrease in aromaticity upon dimerization for all (C_6_H_6_)_2_ conformers but the sandwich one, for which a noticeable increase of 0.0239 is predicted. Again, for asymmetric H-bonded benzene dimers, the drop in aromaticity is lower for the acceptor ring A, than it is for the donor rings. The values of ΔSAI and ΔnMCBO are sensitive to any symmetry breaking or anisotropy in the electron density. Hence, the formation of a benzene dimer from perfectly symmetric (D_6h_) benzene has to lead to a system with slightly reduced aromaticity. The values of NICS(0) suggest that π–π stacking, in either parallel-displaced or sandwich mode, leads to an increase in the aromaticity. The nominal values decrease by ample 1.137 and 0.764 units for the former and the latter isomer, respectively. The magnetic criterion also suggests an overall increase in aromaticity of the T-shaped isomer, although the effect is much smaller as the values of −0.013 and −0.409 were found for rings A and B, respectively. For the most stable T-shaped tilted isomer, the NICS(0) values indicate opposite trends in the aromaticity change. Namely, ring A shows a decrease, whereas ring B shows an increase. The discrepancies between SAI/nMBO and NICS(0) trends arise from the fact that the latter is affected by through-space ring currents from the partner.

In the case of pyridine dimers, ΔHOMA indices suggest a small decrease in aromaticity for all studied motifs. The highest reduction is observed for the H-bonded system whereas the lowest for the T-shaped tilted one. The SAI values exhibit an opposite trend, with aromaticity increasing in all cases, in the following order stacked < T-shaped tilted < H-bonded. For the asymmetric T-shaped tilted isomer, the increase in aromaticity is higher for ring A, which acts as a C–H···π noncovalent interaction donor, than it is for ring B, which donates C–H···N. The Δ*H* values indicate a decrease in aromaticity for all rings across all conformers of the pyridine dimer, consistent with the ΔHOMA results. The values of ΔnMBO calculated for (C_6_H_5_N)_2_ suggest an increase in aromaticity for the H-bonded and a decrease in aromaticity for the stacked conformers, respectively. For pyridine dimers, ΔNICS(0) indicates the opposite trend in aromaticity compared with ΔnMBO. Namely, a 0.232 ppm increase in NICS(0) was calculated for H-bonded pyridine, whereas for the T-shaped tilted isomer, values of 0.088 and 0.350 ppm were calculated for rings A and B, respectively. On the other hand, a −1.680 ppm drop in value was obtained for the stacked isomer. These mixed trends observed in this case illustrate that a small change in NICS(0) does not map one-to-one into the redistribution of electron density. In pyridine, N–H···π and nitrogen atom polarization mainly perturb the local electron density around the nitrogen and neighboring bonds, which is picked up more efficiently by SAI and nMCBO than by the HOMA index.

The values of the ΔHOMA indices calculated for the dimers of furan indicate the largest structural responses to dimerization of all rings considered in this study. This is likely caused by the fact that it has the lowest intrinsic aromatic stabilization (see Table S2). As a result, even small electronic perturbations may induce a relatively significant bond length alteration. Namely, a 0.02952 drop in value was predicted upon formation of the H-bonded conformer. On the other hand, creation of the stacked one is associated with a 0.01140 increase in corresponding HOMA value. For the T-shaped isomers, the absolute values are much smaller and indicate a decrease in aromaticity for all rings but B (acceptor in both C–H···O and C–H···π) in the T-shaped tilted variant. The values of ΔSAI suggest an increase in aromaticity for the T-shaped and stacked isomers and a decrease in overall aromaticity of the H-bonded tilted isomers of (C_4_H_4_O)_2_. Similarly to what was observed from ΔHOMA values, for the T-shaped tilted conformer, a decrease and increase in aromaticity is predicted for rings A and B, respectively. As for the changes in the total energy density at RCP, the data in Table 2 indicate a modest enhancement in aromaticity for all of furan’s dimers except the T-shaped one, which seems to preserve the monomeric level. Calculated ΔNICS(0) values agree in trends with ΔnMBO for all furan dimer isomers but for the stacked one. Namely, an increase in aromaticity is predicted for both T-shaped isomers together with a decrease in aromaticity within the H-bonded variant. As for all stacked conformations in this paper, NICS(0) values demonstrate an increase in aromaticity upon dimerization. In the case of furan, it is by an amount of 1.517 ppm. For the furan dimer, the relatively low intrinsic aromaticity of each furan ring makes both structural and electronic indices more sensitive to small perturbations.

When it comes to the dimers of pyrrole, ΔHOMA, ΔSAI, Δ*H*, and ΔnMBO indices are in agreement when it comes to the aromaticity changes for the stacked isomer, as they all predict a decrease in aromaticity upon dimer’s formation. This is a phenomenon opposite to what can be deduced based on the calculated ΔNICS(0) value of −0.773 ppm. For the T-shaped tilted isomer, the slightly positive ΔHOMA and negative ΔNICS(0) values suggest an increase in the aromaticity. The remaining two quantities, i.e., ΔSAI and ΔnMBO, give different and opposite to each other insights. Namely, the ΔSAI value indicates an increase in the aromaticity for ring A and decrease for ring B. The exact opposite trend is observed when looking at ΔnMBO as the corresponding values are equal to −0.0244 and 0.0099, so a decrease and increase in normalized bond order for rings A and B, respectively. The Δ*H* values calculated for both rings of the T-shaped tilted isomer of (C_5_H_5_N)_2_ suggest a decrease in aromaticity. As was the case for the previous systems, magnetic changes upon dimerization were again found to outsize structural and electronic ones. Together, these results underline that different aromaticity descriptors emphasize different aspects of aromaticity, especially in the presence of a strong anisotropic N–H···π interaction.

In order to quantify how consistently the different aromaticity descriptors behave, the Pearson correlation coefficients between all of the indices and between their changes upon dimerization have been computed. These data are collected in color-coded Tables S3 and S4, respectively. It is evident from the analysis of data in Table S3 that HOMA, SAI, *H*, and nMBO calculated for the studied systems are very highly correlated (or anticorrelated) as the corresponding |*r*| value falls in the 0.85–0.96 range. Overall, both rings within a studied dimer have very similar aromaticities across the studied data set (*r* ≈ 0.95–1.00). At the same time, the values of NICS(0) are only moderately tied to the remaining indices, as the corresponding |r| values are in the 0.40–0.78 range. As can be deduced from the analysis of the values in Table S4, changes upon dimerization are coherent, not random noise. Namely, ΔHOMA, ΔSAI, and Δ*H* are significantly correlated (e.g., *r* = −0.786 for ΔHOMA-A vs ΔSAI-A), all pointing in the same aromaticity direction. The ΔnMBO index values correlate moderately with ΔHOMA, Δ*H*, and ΔNICS(0) (*r* up to ∼0.5). The ΔNICS(0)-A and ΔNICS(0)-B are very strongly correlated, with *r* = 0.931. At the same time, their correlation to the changes in other indices is limited. The other interesting thing that comes from the analysis of Table S4 is that A/B correlations are moderate (e.g., *r* = 0.677 for ΔHOMA, 0.334 for ΔSAI), which is consistent with orientation-dependent and thus asymmetric perturbations.

It was also found during the studies that certain changes in aromaticity indices correlated with components of SAPT analysis. Three relations worth mentioning are presented in Figure 3. Namely, plots in Figure 3a,b show a relationship between ΔNICS(0) values calculated for each ring of a dimer and the dispersion contribution to the total interaction energy and the charge-transfer contribution to induction, respectively. The first relation is characterized by the correlation coefficients equal to 0.817 and 0.804 for rings A and B, in that order. This one likely stems from the fact that the π systems within a given dimer, and especially a stacked one, are characterized by large anisotropic polarizability. The proximity between two rings allows for a significant overlap of the π clouds. As a consequence, molecular arrangements that increase dispersion by increasing π-cloud polarizability simultaneously increase the mutual perturbation of the induced ring currents, leading to more significant changes in NICS(0) values. The anticorrelated relation presented in Figure 3b can be explained by referencing Misquitta’s paper that defines the charge transfer as arising from electron tunneling into the shielded nuclear fields of the neighboring monomers.^125^ Naturally, it becomes significant when their charge densities overlap, which also enables delocalized through-space current pathways. The last of the presented relationships correlates the total energy density at the ring center with the interaction energy between the two monomers. This relationship is significantly weaker than in the two previous cases, as the correlation coefficients are equal to 0.458 and 0.628 for rings A and B, respectively. This relation can be elucidated by the fact that when the dimer binds more tightly, the potential energy density at RCP, which stems from stronger π-delocalization, dominates. At the same time, weaker delocalization causes the kinetic energy density to cancel out a larger portion of the potential energy density, leading to less negative or even positive values of *H*.

**Figure 3:**
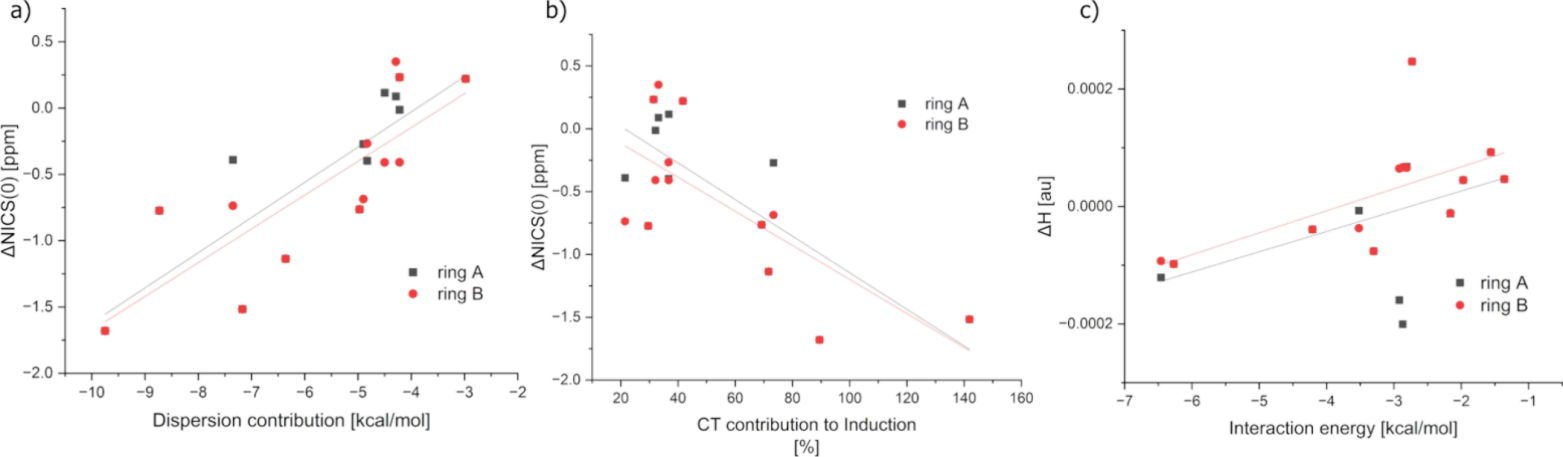
Correlations between SAPT interaction energy components and changes in selected indices upon dimerization: (a) ΔNICS(0) as a function of the SAPT dispersion contribution, (b) ΔNICS(0) versus the charge-transfer contribution to the induction term, and (c) change in the total energy density at the RCP (Δ*H*) as a function of the total interaction energy. The corresponding Pearson’s correlation coefficients for rings A and B are equal to (a) 0.817 and 0.804, (b) −0.758 and −0.722, and (c) 0.458 and 0.628, respectively.

Altogether, it can be said that across all studied systems, both structural and electron-delocalization-based indices suggest only modest changes in aromaticity. The highest change in HOMA, equal to 0.02952, was calculated for the H-bonded conformer of the furan dimer. Similar conclusions can be drawn from the analysis of SAI, total energy density at the RCP (H), and nMBO for which maximum Δ values of 0.00242, 0.002, and 0.0619 were obtained. Interestingly, all of the maxima occur for the dimers of furan. Nonetheless, taken together, these are indicative of weak, noncovalent perturbation rather than a fundamental change in π-delocalization. By contrast, the magnetic response to dimerization seems significant. Namely, the ΔNICS(0) values of −1.680 and −1.517 ppm calculated for the T-shaped and stacked isomers of the furan dimer may appear to indicate an aromaticity boost. However, they likely reflect magnetic effects but not necessarily intrinsic aromaticity changes. Given the weak nature of the interactions within the systems studied here, small and sometimes opposite changes in different aromaticity indices are not only possible but expected.^126,37^ This behavior further reflects the multidimensional character of the aromaticity.

Regarding the influence of the binding mode on changes in aromaticity, some conclusions can also be drawn. Namely, as evident from Table 2, π–π stacking consistently pushes NICS(0) values more negative by 0.737–1.680 ppm. Other aromaticity indices are inconclusive in this regard. T-shaped geometries and their tilted variant geometries yield small, asymmetric changes for rings A and B. The two symmetrical H-bonded motifs of pyridine and furan render ΔNICS(0) positive. The corresponding ΔHOMA values also indicate a small decrease in aromaticity of those geometrical arrangements. At the same time, the remaining indices are equivocal.

Additionally, in order to examine whether the NICS change upon dimerization can be described as an additive effect of the NICS(0) values of the rings, two exemplary systems were analyzed. Namely, there is a benzene sandwich and a stacked pyridine dimer. For each of the two dimers, the NICS(0) of each ring within a dimer was compared with the sum of NICS(0) of monomer A in the geometry of a dimer, and NICS­(x) was calculated at the same spatial point in the field of monomer B alone. For benzene, the sum of the monomer contributions reproduces the dimer NICS(0) values within roughly 0.3 ppm. This is indicative of the fact that the 0.8 ppm decrease is largely a through-space effect of the partner ring. For stacked pyridine, the additive model underestimates the dimer NICS(0) by ∼0.5 ppm, thus revealing a modest nonadditive magnetic coupling.

## Conclusions

This work unravels how dimerization modulates aromaticity in benzene, pyridine, furan, and pyrrole by combining high-level wave functions and energy decomposition with complementary aromaticity metrics including structure (HOMA), electron delocalization (SAI and nMCBO), energetics at ring critical points (Δ*H* in RCP), and magnetism (NICS(0)). In all 13 dimers studied, both the interaction energy driving forces and the aromaticity follow generalizable patterns:

i. Dimer stabilization is dominated by the dispersion contribution in most of the studied cases. Namely, it accounts for 36.47–100.00% of the total attractive interaction. The charge-transfer contribution to stabilization is consistently small and does not exceed 0.44 kcal/mol in any example.
ii. Aromaticity indices based on spatial structure and electron density distribution show only modest changes upon dimerization. In particular, the absolute values of ΔHOMA, ΔSAI, Δ*H*, and ΔnMCBO do not exceed 0.0295, 2.42 × 10^–4^, 2.47 × 10^–4^, and 0.0619, respectively. For most of the studied rings and binding modes, these values change by an order of magnitude less. Altogether, it can be said that the π-delocalization within a monomer ring is preserved in the dimer for the most part. Furan turned out the be most responsive to dimerization, which likely arises from the lowest aromatic stabilization of the isolated ring.
iii. The calculated values of ΔNICS(0) indicate a strong magnetic response to the formation of dimers. The NICS(0) becomes more negative for all π–π bonded systems. At the same time, the remaining dimers show mixed shifts. Because the observed magnetic changes are significantly larger than corresponding structural and electronic ones, they likely reflect through-space coupling between the two aromatic systems rather than significant intrinsic changes in aromaticity.

## Supplementary Material



## Data Availability

The data underlying this study are available in the published article and its Supporting Information.

## Notes

Cartesian coordinates of studied dimers; aromaticity indices of the monomers; correlation coefficients between aromaticity indices; and correlation coefficients between changes in aromaticity indices (PDF)
