# Supplementary material for: Influence of Dimerization on Aromaticity in Benzene and Heteroaromatic Rings
Source: J Org Chem. 2026 Jan 13;91(4):1634–44. doi: 10.1021/acs.joc.5c02533 (PMC13362195; doi:10.1021/acs.joc.5c02533)
Supplement: Supplementary file 1 [file jo5c02533_si_001.pdf]

Supporting Information

Influence of Dimerization on Aromaticity in Benzene and Heteroaromatic Rings

Jakub Brzeski[1,2,*]

[1] Faculty of Chemistry, University of Gdańsk,

Wita Stwosza 63, 80-308 Gdańsk, Poland

[2] QSAR Lab Ltd., Trzy Lipy 3, 80-172, Poland

Table S1 Cartesian coordinates of the studied dimers. 2

Table S2 Aromaticity indices of the monomers 5

Table S3 Correlation coefficients between aromaticity indices 5

Table S4 Correlation coefficients between changes in aromaticity indices upon dimerization .6

* corresponding author: jakub.brzeski@ug.edu.pl

Table S1. Cartesian coordinates in Å of the studied systems* together with corresponding DF-CCSD(T)/aug-cc-pVDZ level energies (in Hartree). Harmonic vibrational analysis confirms that none of the optimized geometries has any imaginary frequency.

Aromatic ring	Dimer geometry	Cartesian coordinates			
pyridine	H-bonded E= -495.2777878	6	-1.339599098	2.962374214	0.000000000
		6	-0.592850041	4.137665300	0.000000000
		6	0.795542057	4.046255291	0.000000000
		6	1.373506099	2.780885203	0.000000000
		6	0.546214042	1.659498122	0.000000000
		7	-0.795542057	1.736597126	0.000000000
		1	-2.422727177	3.001051218	0.000000000
		1	-1.092439080	5.096977370	0.000000000
		1	1.409439102	4.937365357	0.000000000
		1	2.447907177	2.655949191	0.000000000
		1	0.966739067	0.659314047	0.000000000
		6	-0.546214042	-1.659498122	0.000000000
		6	0.592850041	-4.137665300	0.000000000
		6	-1.373506099	-2.780885203	0.000000000
		6	-0.795542057	-4.046255291	0.000000000
		6	1.339599098	-2.962374214	0.000000000
		7	0.795542057	-1.736597126	0.000000000
		1	-2.447907177	-2.655949191	0.000000000
		1	-1.409439102	-4.937365357	0.000000000
		1	1.092439080	-5.096977370	0.000000000
		1	2.422727177	-3.001051218	0.000000000
		1	-0.966739067	-0.659314047	0.000000000
	T-shaped tilted E= -495.2769518	6	1.530321548	0.624357953	-0.149569744
		6	2.659278631	1.432520014	-0.257276752
		6	3.915642720	0.847901971	-0.131249743
		6	3.986624725	-0.522612128	0.097285274
		6	2.801207642	-1.247950181	0.189738281
		7	1.583617555	-0.698183143	0.070615272
		1	0.536644480	1.048489983	-0.241474751
		1	2.549623622	2.493634092	-0.435186765
		1	4.815075786	1.444187013	-0.208775749
		1	4.938407799	-1.025126162	0.202873281
		1	2.823837642	-2.316703256	0.367299293
		6	-2.742746759	0.212563925	1.335310365
		6	-2.758984764	-0.355228116	-1.324651831
		6	-1.645111682	-0.446261123	0.786591325
		6	-1.651259679	-0.737513141	-0.574546774
		6	-3.808068838	0.302147932	-0.684548785
		7	-3.816547840	0.590588951	0.624802314
		1	-0.797919619	-0.723957144	1.399000366
		1	-0.803982619	-1.241522181	-1.020274808
		1	-2.815738764	-0.558172130	-2.385819906
		1	-4.682933898	0.613363952	-1.243618823
		1	-2.767667763	0.451873943	2.392199439

	Stacked<br>E= -495.2801069	6	-2.338202171	-0.253085018	1.054850075
		6	-2.116813154	1.076719076	0.703633049
		6	-1.491378108	1.354315099	-0.508305037
		6	-1.120346083	0.285935021	-1.318255094
		6	-1.385676101	-1.009423071	-0.879650066
		7	-1.983047142	-1.293984093	0.286979021
		1	-2.824173204	-0.499339036	1.992162146
		1	-2.427215177	1.871189136	1.369333097
		1	-1.289649094	2.373328174	-0.810909057
		1	-0.627944044	0.448027033	-2.267484162
		1	-1.105904080	-1.861014137	-1.489348110
		6	2.116813154	-1.076719076	-0.703633049
		6	1.491378108	-1.354315099	0.508305037
		1	2.427215177	-1.871189136	-1.369333097
		6	2.338202171	0.253085018	-1.054850075
		1	1.289649094	-2.373328174	0.810909057
		6	1.120346083	-0.285935021	1.318255094
		7	1.983047142	1.293984093	-0.286979021
		6	1.385676101	1.009423071	0.879650066
		1	2.824173204	0.499339036	-1.992162146
		1	0.627944044	-0.448027033	2.267484162
		1	1.105904080	1.861014137	1.489348110
furan	T-shaped<br>E= -458.9068767	6	-1.050370944	-1.452487115	0.000000000
		6	-2.392512040	-1.933567153	0.000000000
		6	-3.192859100	-0.827160069	0.000000000
		8	-2.437780047	0.304078011	0.000000000
		6	-1.134347949	-0.089128018	0.000000000
		1	-0.138712879	-2.024095156	0.000000000
		1	-2.727203066	-2.956485228	0.000000000
		1	-4.257936176	-0.679941059	0.000000000
		1	-0.404102898	0.701214037	0.000000000
		6	2.055102281	1.751030118	0.712874051
		6	2.055102281	1.751030118	-0.712874051
		6	2.055102281	0.439268021	-1.093213081
		8	2.057240279	-0.370646038	0.000000000
		6	2.055102281	0.439268021	1.093213081
		1	2.052277279	2.601299178	1.372668099
		1	2.052277279	2.601299178	-1.372668099
		1	2.050485279	-0.059282015	-2.045838150
		1	2.050485279	-0.059282015	2.045838150
	H-bonded<br>E= -458.9049850	6	0.316425023	3.784830273	0.000000000
		6	1.394743103	2.851740206	0.000000000
		6	0.828376061	1.610236118	0.000000000
		8	-0.531320036	1.713949123	0.000000000
		6	-0.828376061	3.042233220	0.000000000
		1	0.373677027	4.859444351	0.000000000
		1	2.449645175	3.064598220	0.000000000
		1	1.217391089	0.607355044	0.000000000
		1	-1.874799136	3.288921239	0.000000000
		6	-0.316425023	-3.784830273	0.000000000
		6	-1.394743103	-2.851740206	0.000000000
		6	-0.828376061	-1.610236118	0.000000000
		8	0.531320036	-1.713949123	0.000000000
		6	0.828376061	-3.042233220	0.000000000

		1	-0.373677027	-4.859444351	0.000000000
		1	-2.449645175	-3.064598220	0.000000000
		1	-1.217391089	-0.607355044	0.000000000
		1	1.874799136	-3.288921239	0.000000000
	T-shaped tilted E= -458.9059462	6	-1.039111153	-0.272626206	-0.532997305
		6	-1.581557193	1.028483887	-0.319004291
		6	-2.862496289	0.842091874	0.115767740
		8	-3.150903309	-0.485561221	0.186613745
		6	-2.032703224	-1.151881269	-0.212347284
		1	-0.049135083	-0.524122224	-0.874538330
		1	-1.091239160	1.975074959	-0.466776302
		1	-3.655901343	1.508777922	0.402424761
		1	-2.103313234	-2.224792348	-0.209007284
		6	2.213108083	1.147999897	-0.333278293
		6	2.639192110	-0.046444189	-0.983303342
		6	2.411998093	-1.062222265	-0.097466276
		8	1.876756054	-0.575899229	1.053574810
		6	1.756364050	0.768538872	0.897091795
		1	2.237284080	2.152703971	-0.718717321
		1	3.058490144	-0.147894197	-1.969446413
		1	2.574838108	-2.124731339	-0.126775278
		1	1.333288019	1.300674910	1.730044858
	Stacked E= -458.9069942	6	1.405813100	1.493339107	1.137594081
		6	1.576895115	0.984068072	-0.182284013
		6	0.457402033	1.344715099	-0.875847065
		8	-0.391746029	2.045129147	-0.076907006
		6	0.194544014	2.125260156	1.146995085
		1	2.083086153	1.409446103	1.970188145
		1	2.407061176	0.419193031	-0.569368039
		1	0.126572009	1.184320083	-1.885713136
		1	-0.359362026	2.659254194	1.898286139
		6	-1.405813100	-1.493339107	-1.137594081
		6	-1.576895115	-0.984068072	0.182284013
		6	-0.457402033	-1.344715099	0.875847065
		8	0.391746029	-2.045129147	0.076907006
		6	-0.194544014	-2.125260156	-1.146995085
		1	-2.083086153	-1.409446103	-1.970188145
		1	-2.407061176	-0.419193031	0.569368039
		1	-0.126572009	-1.184320083	1.885713136
		1	0.359362026	-2.659254194	-1.898286139
pyrrole	T-shaped tilted E= -419.2582156	6	-0.771458000	2.298378000	0.709512000
		6	0.476247000	1.859775000	1.122855000
		7	1.214693000	1.609386000	0.000000000
		6	0.476247000	1.859775000	-1.122855000
		6	-0.771458000	2.298378000	-0.709512000
		1	-1.578446000	2.594070000	1.358794000
		1	0.886629000	1.709016000	2.106281000
		1	0.886629000	1.709016000	-2.106281000
		1	-1.578446000	2.594070000	-1.358794000
		1	2.137984000	1.209276000	0.000000000
		6	0.205618000	-2.889183000	0.708296000
		6	-0.282705000	-1.659345000	1.120238000
		7	-0.577152000	-0.934472000	0.000000000
		6	-0.282705000	-1.659345000	-1.120238000

		6	0.205618000	-2.889183000	-0.708296000
		1	0.518994000	-3.689537000	1.357630000
		1	-0.445497000	-1.251077000	2.103016000
		1	-0.445497000	-1.251077000	-2.103016000
		1	0.518994000	-3.689537000	-1.357630000
		1	-0.896561000	0.025874000	0.000000000
	Stacked E= -419.2577049	6	1.458610000	2.031440000	0.709587000
		6	1.458610000	0.709204000	1.121002000
		7	1.473420000	-0.072961000	0.000000000
		6	1.458610000	0.709204000	-1.121002000
		6	1.458610000	2.031440000	-0.709587000
		1	1.480054000	2.890570000	1.359225000
		1	1.448828000	0.271203000	2.103972000
		1	1.448828000	0.271203000	-2.103972000
		1	1.480054000	2.890570000	-1.359225000
		1	1.306634000	-1.067912000	0.000000000
		6	-1.458610000	-2.031440000	0.709587000
		6	-1.458610000	-0.709204000	1.121002000
		7	-1.473420000	0.072961000	0.000000000
		6	-1.458610000	-0.709204000	-1.121002000
		6	-1.458610000	-2.031440000	-0.709587000
		1	-1.480054000	-2.890570000	1.359225000
		1	-1.448828000	-0.271203000	2.103972000
		1	-1.448828000	-0.271203000	-2.103972000
		1	-1.480054000	-2.890570000	-1.359225000
		1	-1.306634000	1.067912000	0.000000000

* The geometries of benzene dimers were taken from the paper of Hobza et al. (Pitoňák, M.; Neogrády, P.; Řezáč, J.; Jurečka, P.; Urban, M.; Hobza, P., J. Chem. Theory Comput. 2008, 4 (11), 1829–1834)

Table S2. Values of aromaticity indices characterizing benzene, pyridine and pyrrole. NICS(0) values are in ppm whereas the remining ones are dimensionless.

Aromatic ring	HOMA	SAI	H	nMBO	NICS(0)
**benzene**	0.9884	0.00	$8.40\cdot10^{-3}$	0.6122	-7.983
**pyridine**	0.9957	$1.03\cdot10^{-3}$	$7.95\cdot10^{-3}$	0.5750	-6.775
**furan**	0.3016	$2.54\cdot10^{-3}$	$2.61\cdot10^{-3}$	0.4706	-12.624
**pyrrole**	0.9084	$3.01\cdot10^{-4}$	$5.81\cdot10^{-3}$	0.5725	-14.357

Table S3. Pearson correlation coefficient values calculated for the aromaticity indices used in the study.

Aromaticity index	HOMA-A	HOMA-B	SAI-A	SAI-B	H-A	H-B	nMBO-A	nMBO-B	NICS(0)-A	NICS(0)-B
**HOMA-A**	1.000	1.000	-0.937	-0.903	0.961	0.959	0.852	0.914	0.617	0.607
**HOMA-B**	1.000	1.000	-0.936	-0.902	0.962	0.960	0.859	0.917	0.622	0.611
**SAI-A**	-0.937	-0.936	1.000	0.982	-0.886	-0.891	-0.849	-0.886	-0.437	-0.415
**SAI-B**	-0.903	-0.902	0.982	1.000	-0.855	-0.860	-0.852	-0.875	-0.416	-0.395
**H-A**	0.961	0.962	-0.886	-0.855	1.000	0.999	0.880	0.899	0.783	0.774
**H-B**	0.959	0.960	-0.891	-0.860	0.999	1.000	0.880	0.893	0.784	0.772
**nMBO-A**	0.852	0.859	-0.849	-0.852	0.880	0.880	1.000	0.952	0.676	0.661
**nMBO-B**	0.914	0.917	-0.886	-0.875	0.899	0.893	0.952	1.000	0.603	0.598

**NICS(0)-A**	0.617	0.622	-0.437	-0.416	0.783	0.784	0.676	0.603	1.000	0.997
**NICS(0)-B**	0.607	0.611	-0.415	-0.395	0.774	0.772	0.661	0.598	0.997	1.000

Table S4. Pearson correlation coefficient values calculated for the changes in the values of aromaticity indices used in the study.

**Aromaticity index**	**ΔHOMA-A**	**ΔHOMA-B**	**ΔSAI-A**	**ΔSAI-B**	**ΔH-A**	**ΔH-B**	**ΔnMBO-A**	**ΔnMBO-B**	**ΔNICS(0)-A**	**ΔNICS(0)-B**
**ΔHOMA-A**	1.000	0.677	-0.786	-0.164	-0.575	-0.457	-0.154	-0.090	-0.402	-0.478
**ΔHOMA-B**	0.677	1.000	-0.635	-0.202	-0.472	-0.422	0.324	0.104	-0.316	-0.474
**ΔSAI-A**	-0.786	-0.635	1.000	0.334	0.530	0.692	-0.136	-0.237	0.259	0.239
**ΔSAI-B**	-0.164	-0.202	0.334	1.000	-0.026	0.105	-0.418	-0.307	0.046	-0.025
**ΔH-A**	-0.575	-0.472	0.530	-0.026	1.000	0.643	0.214	0.352	-0.042	0.015
**ΔH-B**	-0.457	-0.422	0.692	0.105	0.643	1.000	0.047	-0.189	0.279	0.289
**ΔnMBO-A**	-0.154	0.324	-0.136	-0.418	0.214	0.047	1.000	0.769	0.446	0.505
**ΔnMBO-B**	-0.090	0.104	-0.237	-0.307	0.352	-0.189	0.769	1.000	0.277	0.437
**ΔNICS(0)-A**	-0.402	-0.316	0.259	0.046	-0.042	0.279	0.446	0.277	1.000	0.931
**ΔNICS(0)-B**	-0.478	-0.474	0.239	-0.025	0.172	0.289	0.505	0.437	0.931	1.000
